# Preparation, Characterization, Photochromic Properties, and Mechanism of PMoA/ZnO/PVP Composite Film

**DOI:** 10.3390/molecules28227605

**Published:** 2023-11-15

**Authors:** Tiehong Song, Jinyao Li, Qiyuan Deng, Yanjiao Gao

**Affiliations:** 1 Urban Construction College, Changchun University of Architecture and Civil Engineering, Changchun 130600, China; 2 Key Lab of Songliao Aquatic Environment, Ministry of Education, Jilin Jianzhu University, Changchun 130118, China; ljyjja@163.com (J.L.); 18041778377@163.com (Q.D.); 3 Liao Yuan Vocational Technical College, Liaoyuan 136200, China; 4 College of Civil Engineering and Architecture, Liaoning University of Technology, Jinzhou 121001, China; tmgyj@lnut.edu.cn

**Keywords:** visible light, phosphomolybdic acid, zinc oxide, polyvinylpyrrolidone, photochromic material

## Abstract

A novel photochromic heteropolyacid-based composite film consisting of phosphomolybdic acid (PMoA), ZnO, and polyvinylpyrrolidone (PVP) was fabricated by a sol–gel process. The microstructure and photochromic properties of the PMoA/ZnO/PVP were characterized via Fourier transform infrared spectroscopy (FTIR), transmission electron microscopy (TEM), X-ray photoelectron spectroscopy (XPS), and ultraviolet-visible spectroscopy (UV-Vis). The FTIR spectra showed that the basic structures of ZnO and PVP, and the Keggin structure of PMoA in the PMoA/ZnO/PVP composite film, had not been destroyed during the preparation. The TEM images demonstrated that ZnO presented a rod-like structure, while PMoA was spherical, and many PMoA balls adhered to the surface of the ZnO rods. The XPS spectra of Mo 3d indicated that the valency of Mo atoms in the PMoA/ZnO/PVP was changed by visible light exposure. After visible light irradiation, the PMoA/ZnO/PVP varied from slight yellow to blue, while undergoing an opposite color change upon heating. The discoloration mechanism of the PMoA/ZnO/PVP was consistent with the photoelectron transfer mechanism.

## 1. Introduction

Owing to the requirement to deal with various documents, human beings consume too much printing paper, and this printed paper, in addition to archiving, are generally not necessary to keep, resulting in a large amount of waste paper. If printed paper can be reused, it will reduce the requirements for products from the paper industry, thereby protecting the environment and contributing to the sustainable development of human society. Photochromic materials for printing paper offer the possibility of paper recycling. Photochromic materials refer to the special materials that can change their colors after being excited by various light sources (ultraviolet [1] and visible light [2]) and can return to their original colors under certain conditions [3]. These materials can be applied not only in information storage, molecular detection, optical switches, and photochemical sensors, but also in inkless printing with a great outlook [4,5,6,7,8]. The color-changing properties of this inkless printing paper depend on the chemical composition of the photochromic material used to make the paper and on different light source conditions. In order to improve photochromic performance, the components of the material are critical.

Simple photochromic materials include organic materials and inorganic materials. Organic photochromic materials (spiropyrans, salicylaldehyde anilines, azobenzenes, diarylethenes, etc.) are sensitive to color change and easy to process, but they are not very stable [9,10]. Inorganic photochromic materials (transition metal oxides, transition metal polyacid compounds, etc.) possess favorable stability and fatigue resistance, but low color fading rate [11,12]. Introducing some active ingredients into organic or inorganic photochromic materials is a key way to improve the stability and color-changing properties of photochromic materials. Compared with organic photochromic materials, the doping of inorganic photochromic materials has received more attention. For example, noble metals, Pt, Au, etc., can be used to modify the surface of transition metal semiconductors (MoO_3_ and WO_3_) to reduce the electron-hole recombination rate after illumination, and to improve the photochromic reaction efficiency of the composite materials [13,14,15]. Visible light photochromic materials can also be prepared by compounding photosensitive materials TiO_2_ or ZnO with MoO_3_ or WO_3_ [16,17,18]. However, the production costs of the above-mentioned materials are also very high.

Heteropolyacids are the most common transition metal polyacid compounds employed toward the studies of photochromic materials. These compounds can accept one or more electrons, have stable structures, high lattice oxygen activities and excellent redox reversibilities [19,20]. Keggin-type heteropolyacids (phosphomolybdic acid and phosphotungstic acid) are representative polyacid compounds that are widely used in the preparation of photochromic materials due to their unique structures and high oxidative activities [21,22]. Heteropolyacid-based photochromic materials can improve the charge transfer ability of the heteropoly compounds, thereby reducing the excitation energy required for photochromic reaction of the composite materials [23]. Jing et al. [24] dropped photosensitizer ZnFe_2_O_4_ nanoparticles into a silicon dioxide sol–gel matrix to prepare PMoA/SiO_2_/ZnFe_2_O_4_ composite films with high visible light photochromic responsivity and reversible photochromic properties, and pointed out the photoelectron transfer mechanism in the photochromic process of the composite films. Sun et al. [25] prepared a PMoA/ZnO nanosphere that changed color under visible light and returned to its original color with hydrogen peroxide (H_2_O_2_) oxidation. Another PMoA/ZnO nanotube was also synthesized through a hydrothermal synthesis method and could change color from pale yellow to blue under visible light and return to pale yellow in the presence of H_2_O_2_ [26]. The above two studies all testified to a Zn-O-Mo charge transfer bridge being built between ZnO and PMoA, and pointed out that the photochromic mechanism was based on transfer of a photoinduced electron from ZnO to PMoA. Huang et al. [27] fabricated a PVP/PMoA film via layer-by-layer assembly with ultrasonic pretreatment, and the color of PVP/PMoA film changed from colorless to blue via visible light illumination. Furthermore, the bule color of the PVP/PMoA film could gradually fade when exposed to air or heated. Huang et al. [27] also found that ultrasonic cavitation enhanced the photochromic capabilities of the PVP/PMoA film. The previous studies have provided a good foundation for research into heteropolyacid-type photochromic materials.

In this study, PMoA/ZnO/PVP composite films were papered by the sol–gel method, aiming to construct a heteropolyacid-based multi-component visible light photochromic system with excellent photosensitivity and photochromic properties. By means of FTIR, TEM, XPS, and UV-vis, the composition and structure of PMoA/ZnO/PVP were analyzed in detail. The constructed PMoA/ZnO/PVP can be used to prepare visible light photochromic paper, which may realize the reuse of paper and reduce the consumption of papermaking materials.

## 2. Results and Discussion

### 2.1. Characterization of PMoA/ZnO/PVP (PMOA: ZnO = 7:3, A3)

#### 2.1.1. Analysis of FTIR

**Figure 1:**
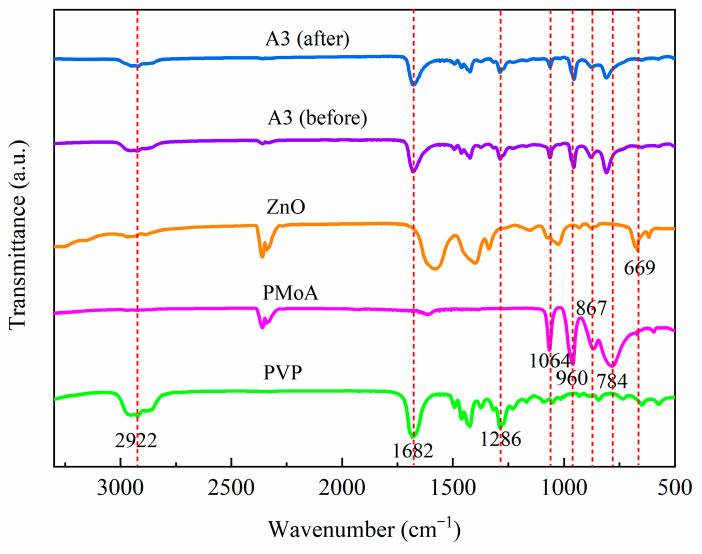
FTIR spectra of PVP, PMoA, ZnO, and PMoA/ZnO/PVP (A3) before illumination and of PMoA/ZnO/PVP (A3) after illumination.

Figure 1 shows the FTIR spectra of PVP, PMoA, ZnO, and PMoA/ZnO/PVP composite film before and after visible light irradiation. The characteristic peaks of PVP were located at 2922 cm^−1^, 1682 cm^−1^, and 1286 cm^−1^, corresponding to C-H, C=O and C-N bonds, respectively [4,16,28]. PMoA had four characteristic peaks at 1064 cm^−1^, 960 cm^−1^, 867 cm^−1^, and 784 cm^−1^, which represented υ(P-O), υ(Mo-Od), υ(Mo-Ob-Mo), and υ(Mo-Oc-Mo) in the Keggin structure, respectively [29,30]. The characteristic peak of ZnO was located at 669 cm^−1^, indicating the υ(Zn-O) bond [31,32]. Comparing the spectra of PMoA/ZnO/PVP A3 (before) with PVP, PMoA, and ZnO, it was found that the characteristic peaks of PVP, PMoA, and ZnO could all be observed in the A3 (before) spectra, which indicated that PVP, PMoA, and ZnO were successfully synthesized into A3 composite film. In addition, according to the A3 (after) spectra, it can be seen that the characteristic peaks of the three materials, PVP, PMoA, and ZnO, still exist after illumination, which testified that the composition of A3 film did not change after illumination. However, compared to the peak positions of Mo-Ob-Mo vibration and Mo-Oc-Mo vibration in the composite film before illumination, the peak positions of Mo-Ob-Mo vibration (867 cm^−1^) and Mo-Oc-Mo vibration (784 cm^−1^) were blueshifted (5 cm^−1^) and redshifted (2 cm^−1^), respectively, after illumination. Also, the characteristic peak of ZnO (669 cm^−1^) underwent a little blue shift (1 cm^−1^). Owing to the strong non-chemical bonding interactions between the two phases of PMoA and ZnO in the PMoA/ZnO/PVP composite film, this shifted the positions of the characteristic peaks of PMoA and ZnO. The study on PMoA/ZnO photochromism by Sun et al. [25] also confirmed that this displacement occurred due to the interaction between PMoA and ZnO. Under the excitation of visible light, the composite film eventuated a photoreduction reaction, and the heteropolyacid molecule accepted photogenerated electrons to generate heteropolyblue [33].

#### 2.1.2. Analysis of TEM

**Figure 2:**
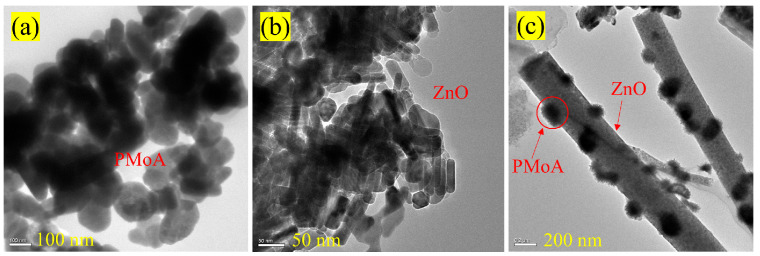
TEM image of PMoA (**a**), ZnO (**b**), and PMoA/ZnO/PVP (**c**).

The TEM patterns of the PMoA, ZnO, and PMoA/ZnO/PVP composite are shown in Figure 2a, Figure 2b, and Figure 2c, respectively. Figure 2a showed that the original PMoA assumed a sphere-like shape with an average diameter of about 50 nm and Figure 2b indicated that ZnO possessed a rod-like structure with a diameter of about 100–160 nm. From Figure 2c, spherical PMoA was found to be bound to the rod-like ZnO surface to form a composite film with the PVP film as the substrate. ZnO was not completely covered by PMoA, which was required for the experimental concentration because the surface of ZnO needed to be excited by light. Building on Figure 1 and Figure 2c reconfirmed that PMoA and ZnO are successfully bound to the PVP, resulting in the formation of a PMoA/ZnO/PVP composite film. Yun et al. [26] introduced PMoA into ZnO nanotubes by hydrothermal synthesis to form a photochromic powder. The SEM and TEM images showed that the ZnO nanotubes exhibited rod and tube structures and a length range of 235–275 nm. And, PMoA nanoparticles were wrapped around the outer surface of the ZnO nanotubes, resulting in roughness, which was caused by the acidic etching effect. The morphology of PMoA/ZnO/PVP in this study is similar to the morphology of the PMoA/ZnO prepared by Yun et al., except that the material composition in this study contains PVP.

### 2.2. Photochromic Properties of Composite Films

**Figure 3:**
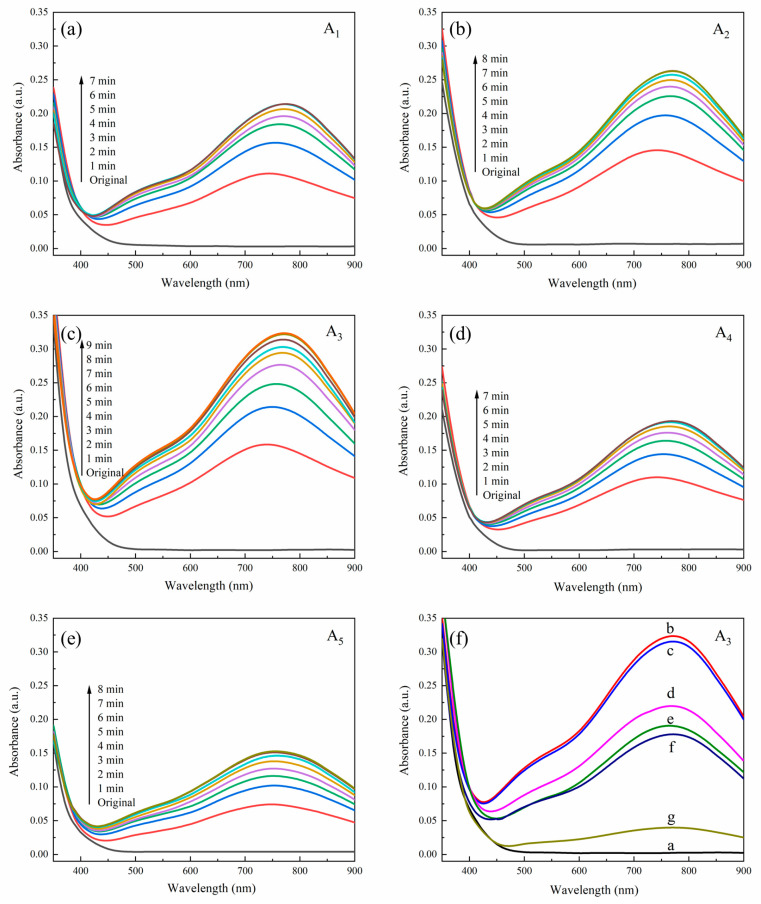
(**a**–**e**) UV-vis spectra of PMoA/ZnO/PVP composite film sample Ai (i = 1, 2, 3, 4, 5); (**f**) bleaching process of A3 composite film after irradiation. (a) Without irradiation; (b) irradiation for 9 min; (c) 3 d under vacuum after irradiation for 9 min; (d) 1 d in oxygen after irradiation for 9 min; (e) 2 d in oxygen after irradiation for 9 min; (f) 3 d in oxygen after irradiation for 9 min; and (g) heating at 100 °C for 30 min after irradiation for 9 min.

The UV-Vis absorption spectra of the composite films Ai (i = 1, 2, 3, 4, 5) before and after irradiation are shown in Figure 3a–e, and the bleaching process of A3 is depicted in Figure 3f. The absorption peak ranges, absorption saturation times and saturation absorbance values of A1, A2, A3, A4, and A5 under visible light irradiation are exhibited in Table 1.

After visible light irradiation, the colors of A1–A3 changed from pale yellow to blue, and all showed new characteristic absorption peaks at 750–800 nm. The newly emerged characteristic peaks could be attributed to the transition peaks of the valence layer charge transfer of the reduction of metallic Mo^6+^ to Mo^5+^, which indicated that photochromic reactions occurred in the prepared composite films under visible light. The doping of ZnO was increasing from A1 to A5, but their light-absorbing capacities were not gradually increasing. When the PMoA:ZnO was 7:3 (in A3), A3 obtained the largest saturation absorbance value of 0.323. When the PMoA:ZnO was greater than 7:3 (in A1 and A2), due to excessive chromophore PMoA, the photosensitizer ZnO was relatively insufficient, and ZnO could not provide sufficient electrons for the reduction reaction of phosphomolybdic acid. When the PMoA:ZnO was less than 7:3 (in A4 and A5), the photosensitizer ZnO was saturated and the chromophore PMoA was relatively insufficient, so that the saturated absorbance decreased. Therefore, the optimum ratio of PMoA:ZnO was determined to be 7:3 when preparing PMoA/ZnO/PVP composite films.

The fading process curves of the colored composite film A3 are shown in Figure 3f. Curve a showed that A3 had no absorption peak without visible light illumination, and in Curve b A3 reached saturation and obtained the highest absorption peak after 9 min of visible light illumination. The colored composite film A3 was kept in a vacuum environment for 3 days, and the light absorption curve remained almost unchanged (Curve c). However, when exposed to air, the color of A3 began to fade slowly, indicating that oxygen played a key role in the color-fading process. When A3 faded in the air for 1 day, 2 days, and 3 days, the saturated absorbance values of A3 dropped to 68%, 59%, and 55% (Curves d–f), respectively, which indicated that the fading rate of the A3 gradually decreased with the fading time. When the A3 was heated to 100 °C for 30 min, the saturated absorbance value of the A3 rapidly decreased to 12% (Curve g), which indicated that heating accelerated the fading rate of the colored composite film A3. To sum up, the pigmented composite film A3 can be oxidized under air conditions, resulting in a fading reaction, and heating can accelerate the fading rate of the composite film. It was confirmed that the PMoA/ZnO/PVP composite film (A3) prepared in this experiment had reversible photochromic properties. Zhang et al. [34] developed a TiO_2_/PMoA/PVP rewritable paper that can change from light yellow to black-green under UV light, and the color of the paper can be gradually restored to its original light yellow color after 6 min of ozone (3 wt%) treatment. ZnO and TiO_2_ are both photoresponsive semiconductors. ZnO is a visible light catalyst and TiO_2_ is a UV catalyst. When both of them are made into composites together with PMoA, the composites both undergo a similar photochromic reaction, which is based on the transfer of electrons between Mo^6+^ and Mo^5+^. Wang et al. [35] doped TiO_2_ in P_2_W_16_Mo_2_/PVA(polyvinylalcohol) solution to form photochromic composite films. Under UV irradiation, the photochromic response time of the TiO_2_-doped composite films was more sensitive and the UV-visible light adsorption increased significantly. Moreover, the P_2_W_16_Mo_2_/PVA/TiO_2_ composite film could return to its original color in the presence of oxygen. Mo^5+^ was oxidized to Mo^6+^ during the fading process, which was similar to the mechanism of PMoA/ZnO/PVP bleaching.

**Figure 4:**
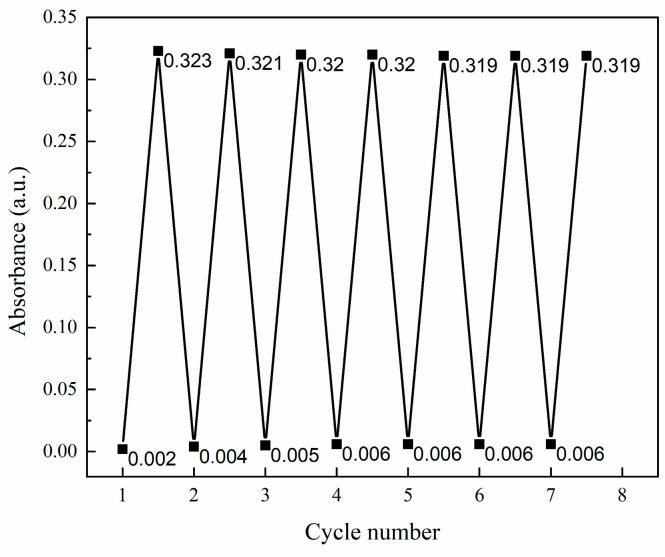
Coloring—discoloration cycle diagram for PMoA/ZnO/PVP composite film.

Figure 4 shows the change in light absorption properties of composite film A3 after coloring-fading cycles. The saturated absorbance of the composite film A3 was almost unchanged in seven coloring-fading cycle experiments, which indicated that A3 has excellent, stable and reversible photochromic properties. The composite film was still firmly adhered to the substrate after seven cycles of experiments, indicating that the obtained film had good adhesion and stable physical properties.

### 2.3. Photochromic Mechanism of Composite Films

**Figure 5:**
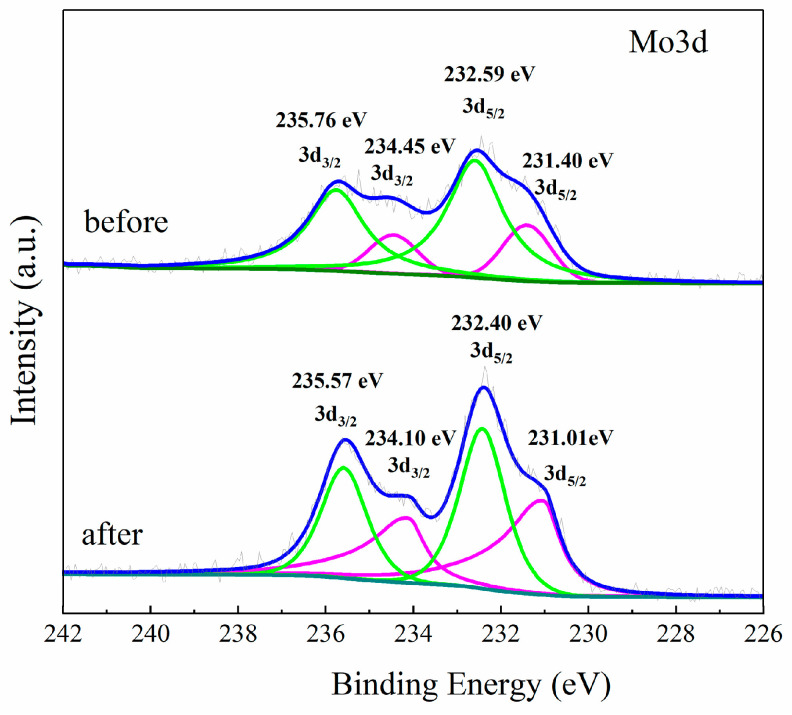
XPS spectra of Mo 3d (before illumination and after illumination).

Figure 5 depicts the XPS spectra of Mo 3d in the PMoA/ZnO/PVP composite film before and after illumination. The binding energy of Mo 3d can be decomposed into spin-orbit Mo 3d5/2 and Mo 3d3/2 by Gaussian solution [26,36,37]. The characteristic signals of Mo^5+^ and Mo^6+^ are both found in Figure 5 (before illumination and after illumination). Before illumination, the peaks of Mo^5+^ appeared at 231.40 eV and 234.45 eV, and Mo^6+^ achieved maximum peak positions at 232.59 eV and 232.76 eV. Mo^5+^ was detected in film without irradiation via visible light due to the bombardment of Mo^6+^ by X-rays from the XPS instrument, and can be considered as a background value. The same test conditions (5 scans, 1 m 40.2 s, 500 μm, and CAE20.0, 0.05 eV) were used for the XPS test of the visible light-exposed film, which made the results comparable and reliable. In Figure 5, compared with before illumination, the binding energies of Mo 3d5/2 and Mo 3d3/2 changed slightly after illumination, which indicated that the chemical microenvironment of Mo atoms in the composite films changed after illumination. Mo^5+^/Mo increased from 0.24 (before illumination) to 0.48 (after illumination), indicating that, after subtracting the X-ray produced Mo^5+^, visible light irradiation actually reduced Mo^6+^ in the composite film to form Mo^5+^. In the study of Zhang et al. [34], a significant enhancement of the Mo^5+^ signal in the TiO_2_/PMoA composite nanoparticles after UV illumination was observed, which indicated that Mo^6+^ was reduced to Mo^5+^ by photogenerated electrons upon UV illumination. This appearance is consistent with this study.

**Figure 6:**
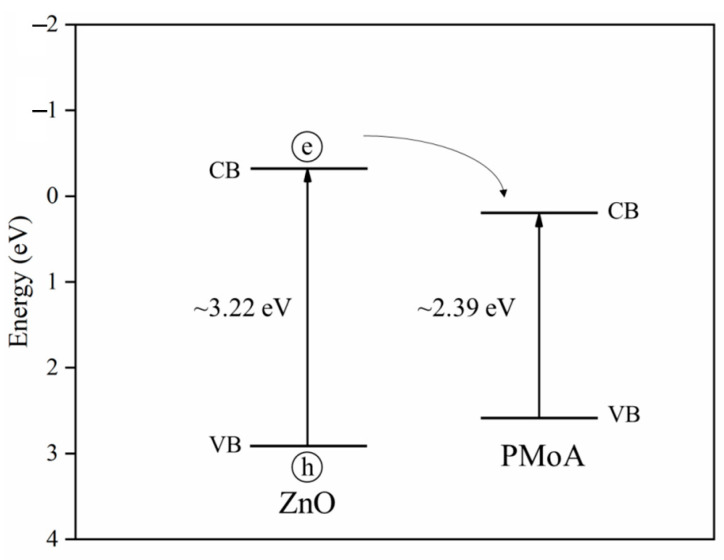
Bandgaps of ZnO and PMoA.

**Figure 7:**
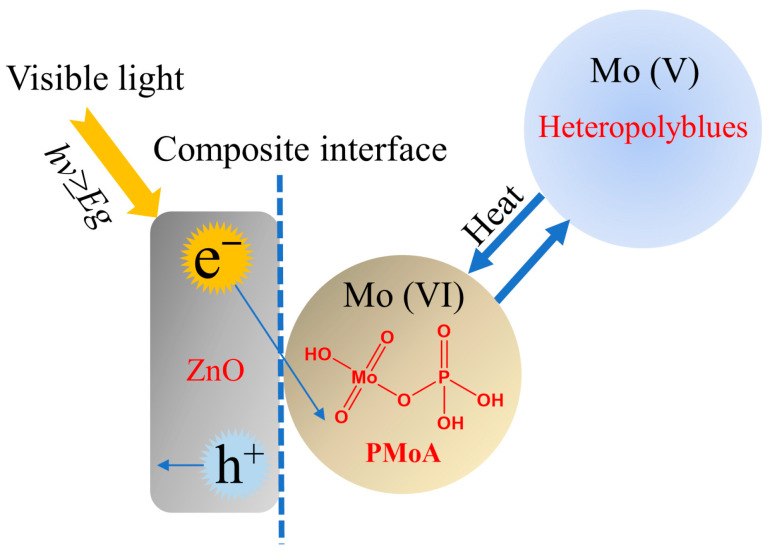
Diagram of color change mechanism of PMoA/ZnO/PVP composite film.

In order to analyze the photocatalytic mechanism, the positions of valence and conduction bands of ZnO and PMoA are depicted in Figure 6 based on previous studies [26,38,39]. The bottom of the valence band of ZnO was significantly lower than that of PMoA, indicating that electrons in ZnO could easily enter into PMoA. The conduction band position of PMoA was lower than that of ZnO, which was conducive to the injection of excited electrons into the conduction band of PMoA. In this reaction, ZnO acted as an electron donor and donated electrons to PMoA under visible light illumination, and so PMoA obtained electrons and underwent a reduction reaction, and Mo_6+_ was reduced to Mo^5+^ to generate heteropolyblue [21,22], which is consistent with the conclusion drawn by XPS. Therefore, the photochromic mechanism of the PMoA/ZnO/PVP composite film was based on the electron transfer mechanism. The mechanism of photochromism and the bleaching process of the composite film are shown in Figure 7. ZnO serves as an electron donor providing electrons under the illumination of visible light to deliver electrons to PMoA. Then, PMoA receives electrons and forms heteropolyblues, resulting in a color change in the film (from slight yellow to blue). Conversely, heat is used for treating the heteropolyblue, leading to a bleaching process to restore the original color.

## 3. Materials and Methods

### 3.1. Materials

Phosphomolybdic acid (PMoA, H_3_[PMo_12_O_40_]•xH_2_O) was purchased from Sinopharm Chemical Reagent Co., Ltd. (Runcorn, UK) and recrystallized twice. Polyvinylpyrrolidone (PVP, K90) was obtained from Tianjin Fengchuan Chemical Reagent Technology Co., Ltd. (Liqizhuang, Tianjin, China) and purified by fractional distillation. Zinc acetate (Zn(CH_3_COO)_2_) and ethanolamine (C_2_H_7_NO) were purchased from Sinopharm Chemical Reagent Co., Ltd. (Shanghai, China) Anhydrous ethanol (C_2_H_6_O) and acetone (CH_3_COCH_3_) were purchased from Beijing Chemical Plant. All other chemical reagents were analytical grade, and deionized water was used in all experiments.

### 3.2. Preparation

For preparing ZnO, zinc acetate dihydrate (8.23 g) was dissolved in anhydrous ethanol (262.75 mL) and stirred in a water bath (60 °C) for 0.5 h. Slowly, 2.25 mL of ethanolamine was added dropwise to the stirred solution and stirring continued in a water bath (60 °C) until a homogeneous and transparent solution was formed; this was then left for 24 h to form a zinc oxide sol.

A drop-coating method was used to prepare thin film samples. The quartz glass was used as the substrate, the size of the substrate was 1.5 cm × 2.0 cm and the thickness was about 1 mm. The oxidizer used to modify the substrate was Piranha detergent. The preparation method for the Piranha detergent was as follows: mix 98% of concentrated sulfuric acid and 30% of hydrogen peroxide in a volume ratio of 7:3 and cool to room temperature for later use.

The concentration ratios of PMoA:ZnO were designed as 9:1, 8:2, 7:3, 6:4, and 5:5. Under the condition of constant stirring, ZnO sol solution was slowly added dropwise to the PMoA ethanol solution for preparing PMoA/ZnO. The mixed PMoA/ZnO solution was magnetically stirred for 30 min, then subjected to ultrasound for 30 min, and finally magnetically stirred for 60 min. In the absence of light, 100 μL of PMoA/ZnO mixture was slowly added dropwise to 10 mL of PVP ethanol solution, and the PMoA/ZnO/PVP mixture was first magnetically stirred for 30 min, then shaken by ultrasonic waves for 60 min, and finally magnetically stirred for 60 min. Up to this point, a homogeneous and transparent light yellow complex solution Ai (i = 1, 2, 3, 4, 5) was obtained. A volume of 100 μL of complex solution Ai was applied evenly onto the substrate by dropping, and allowing it to dry naturally to form a film.

### 3.3. Instrumental Analysis

A transmission electron microscope (TEM, ASAP2010M, Shanghai, China) with a resolution of 0.2 nm was used to observe the fine structure of the composite films. An ultraviolet–visible absorption spectroscope (UV-Vis, JASCO v-550, Shanghai, China) with an optical resolution of 1 nm and a wavelength range of 350–900 nm was utilized to analyze the absorbance curves of the films. An X-ray photoelectron spectroscope (XPS, ESCALAB 250, Shanghai, China) and a Fourier transform infrared spectrometer (FTIR, Nicolet 550, Shanghai, China) were used to detect the valence band and structure of the composite films. A xenon lamp (75 W, 15 V, 5 A, λ > 422 nm) was employed as the visible light source in the photochromic performance experiment. The distance between the film and the light source was 15 cm. Experiments were performed at room temperature.

## 4. Conclusions

In summary, PMOA/ZnO/PVP composite film was successfully developed as an effective photochromic material that responds to visible light. In the PMOA/ZnO/PVP composite film, PVP acted as a basement membrane with rod-shaped ZnO and spherical PMOA inlaid onto it. The basic structures of PVP, ZnO, and PMoA in the composite films were not destroyed during the composite process or visible light illumination. The transfer of non-covalent bond Zn-O-Mo was built between ZnO and PMoA, which greatly improved photochromic reaction rate. The composite film changed from light yellow to blue under visible light, returning to its original color upon exposure to air or heat. The photoreduction of PMoA took place after Mo^6+^ was reduced to Mo^5+^, thus heteropolyblue was formed. Therefore, the photochromic mechanism of PMoA/ZnO/PVP composite film that occurred was based on the electron transfer.

## Figures and Tables

**Table 1:** Light absorption properties of different composite films.

Composite Film	Peak Range (nm)	Saturation Time (min)	Absorbance Value
A1	765–780	7	0.214
A2	758–778	8	0.262
A3	761–782	9	0.323
A4	751–780	7	0.193
A5	742–773	8	0.153

## Data Availability

Data is contained within the article.
